# Short peptide based nanotubes capable of effective curcumin delivery for treating drug resistant malaria

**DOI:** 10.1186/s12951-016-0179-8

**Published:** 2016-04-05

**Authors:** Shadab Alam, Jiban Jyoti Panda, Tapan Kumar Mukherjee, Virander Singh Chauhan

**Affiliations:** International Centre for Genetic Engineering and Biotechnology, New Delhi, 110067 India; Institute of Nano Science and Technology, Mohali, Punjab 160062 India; Maharishi Markandeshwar University, Ambala, Haryana 133207 India

**Keywords:** Nanotubes, Antimalarial, Self-assembly, Peptide, Curcumin

## Abstract

**Background:**

Curcumin (Ccm) has shown immense potential as an antimalarial agent; however its low solubility and less bioavailability attenuate the in vivo efficacy of this potent compound. In order to increase Ccm’s bioavailability, a number of organic/inorganic polymer based nanoparticles have been investigated. However, most of the present day nano based delivery systems pose a conundrum with respect to their complex synthesis procedures, poor in vivo stability and toxicity issues. Peptides due to their high biocompatibility could act as excellent materials for the synthesis of nanoparticulate drug delivery systems. Here, we have investigated dehydrophenylalanine (ΔPhe) di-peptide based self-assembled nanoparticles for the efficient delivery of Ccm as an antimalarial agent. The self-assembly and curcumin loading capacity of different ΔPhe dipeptides, phenylalanine–α,β-dehydrophenylalanine (FΔF), arginine-α,β-dehydrophenylalanine (RΔF), valine-α,β-dehydrophenylalanine (VΔF) and methonine-α,β-dehydrophenylalanine (MΔF) were investigated for achieving enhanced and effective delivery of the compound for potential anti-malarial therapy.

**Results:**

FΔF, RΔF, VΔF and MΔF peptides formed different types of nanoparticles like nanotubes and nanovesicles under similar assembling conditions. Out of these, F∆F nanotubes showed maximum curcumin loading capacity of almost 68 % W/W. Ccm loaded F∆F nanotubes (Ccm-F∆F) showed comparatively higher (IC_50_, 3.0 µM) inhibition of *Plasmodium falciparum (Indo strain)* as compared to free Ccm (IC_50_, 13 µM). Ccm-F∆F nano formulation further demonstrated higher inhibition of parasite growth in malaria infected mice as compared to free Ccm. The dipeptide nanoparticles were highly biocompatible and didn’t show any toxic effect on mammalian cell lines and normal blood cells.

**Conclusion:**

This work provides a proof of principle of using highly biocompatible short peptide based nanoparticles for entrapment and in vivo delivery of Ccm leading to an enhancement in its efficacy as an antimalarial agent.

## Background

Malaria, one of the most devastating infectious disease, affects almost half of the global population [1]. In humans, malaria is caused by a unicellular organism, the *Plasmodium* parasite and is transmitted through the female *Anopheles* mosquito. *Plasmodium* parasite has many different species, of which *P. falciparum* accounts for most human deaths mostly in sub Saharan Africa. Malaria symptoms are generally associated with headache, chills, fever and vomiting, which are initially mild and hence difficult to distinguish as malaria. If appropriate treatment is not administered within 24 h, *P. falciparum* infection can progress and cause severe anaemia, respiratory distress or cerebral malaria which often leads to death [2].

In the present scenario, few drugs like chloroquine, sulfadoxine–pyrimethamine (SP), artemisinin and its derivatives are the only available effective treatment modalities for malaria. However, drug resistance and toxicity to most of the available drugs like chloroquine and SP poses a serious and growing challenge to treat malaria [3–7] Artemisinin and its combinations with other drugs (ACT’s) have been successfully used to treat malaria, but recent reports of resistance to artemisinin particularly in many Southeast Asian countries has made the situation grim [8, 9]. Therefore, there is an urgent need to develop new drugs and new treatment strategies to cure malaria. Several chemical compounds either synthetic or isolated from natural sources are being actively investigated for their antimalarial activity [10–14] and although there seems to be a fairly good number of anti-malarial molecules in pipeline, the situation is far from satisfactory. Curcumin (Ccm) isolated from the rhizomes of curcuma longa (turmeric), has been shown to possess strong antimalarial activity in several studies. It has been shown to disrupt microtubules, inhibit histone acetylation and generate reactive oxygen species to kill the parasite [15–17]. However, certain features like poor absorption, rapid metabolism and fast elimination from the body lead to its low bioavailability and limit its therapeutic effects as an anti-malarial agent [18].

Nanoparticles owing to their small size demonstrate special features like protection of drugs from non-specific degradation, increment in drug half-life, prevention of drug resistance, increased bioavailability, site specific delivery of therapeutic compounds and reduced toxicity to other body parts etc [19–21]. A number of organic/inorganic polymer based nanoparticles have been developed as efficient drug delivery systems and many of these have also been used for the entrapment and delivery of Ccm [22–24]. However, most of these nanoparticles demonstrate low biocompatibility, which prevent their direct applications in human targets [25–29].

Moreover, the complex way of synthesis of these nanoparticles poses hurdles in their large scale synthesis and commercialization. In this context nanoparticles based on biocompatible building blocks offer an attractive alternative and in recent years a number of peptide based nanoparticles have been reported [30–33]. But, on the other hand peptide based systems also have an inherent drawback in that they are susceptible to enzymatic degradation leading to their short half-life in in vivo situations. In this regard short peptide based nanoparticles; particularly those containing unnatural amino acids may offer an attractive alternate solution. In this direction we have used α, β-dehydroamino acids in designing short peptides which can self-assemble into stable and highly biocompatible nanostructures and as potential biomolecule delivery system [34–36].

Here, we report synthesis and characterization of α,β-dehydrophenylalanine containing self-assembling and biocompatible dipeptide nanoparticles (DNPs) and their potential as drug delivery systems for hydrophobic drugs like Ccm. Results of both in vitro and in vivo studies demonstrated enhanced antimalarial activity with DNPs loaded Ccm in comparison to the free drug. The unique one step synthesis, long term stability of these DNPs and their biocompatibility make them highly effective platforms for further development as efficient carriers for hydrophobic drug like Ccm.

## Results and discussion

### Synthesis and characterisation of the DNPs

Four different dipeptides used in the present study were synthesised using solution phase peptide synthesis methods. The peptides were purified by reverse phase high performance liquid chromatography (RPHPLC) and characterised using mass spectrometry (Table 1).

**Table 1:** Characterization of dipeptides: HPLC retention time and mass of FΔF, RΔF, MΔF and VΔF dipeptide

S.No.	Nanostructure	HPLC retention time (min)	Expected mass	Observed mass
1	RΔF	28.0	319.39	320.181
2	MΔF	29.5	294.407	295.118
3	VΔF	34.5	262.341	263.342
4	FΔF	24.0	310.38	311.140

For initiating self-assembly, the peptides (2 mg each in case of R∆F, V∆F and M∆F and 0.5 mg in case of F∆F) were first dissolved in 100 µl of isopropanol followed by addition of 1 ml of water. The samples were further incubated for 5–6 h at room temperature. Formation and properties of the DNPs were then studied using dynamic light scattering (DLS) and transmission electron microscopy (TEM). From light scattering studies, it was observed that all the four dipeptides could form monodispersed nanostructures with low polydispersity indices. RΔF formed nanoparticles with hydrodynamic diameter of 304 ± 20 nm, whereas MΔF, VΔF, and FΔF formed nanoparticles of hydrodynamic diameter of 200 ± 15, 220 ± 25 and 980 ± 45 nm respectively (Table 2). It is likely that dipeptides self assembled under these conditions by virtue of nanoprecipitation mechanism, where isopropanol acted as a solvent and water as an anti-solvent [37]. Hydration with water perhaps renders the hydrophobic moieties of the peptides insoluble, triggering the self-assembly process. Assembly could be stabilized by non-covalent interactions like hydrogen bonding, hydrophobic interactions, van der Waals and electrostatic interactions as well as π–π stacking interactions between the aromatic residues [38, 39]. Head to tail hydrogen bonding and π–π stacking interactions responsible for stabilizing the assembled nanotubes were observed in crystal structure of FΔF [34]. This is in line with the self-assembly of Phe–Phe, where the dipeptide was first dissolved in an organic solvent like in hexafluoroisopropanol at a concentration of 100 mg/ml and then diluted with water to a final concentration of 0.5 mg/ml to form nanotubes [40].

**Table 2:** Characterization of DNPs: hydrodynamic diameters and polydispersity indices of dipeptide nanoparticles

S.No.	Nanostructure	Hydrodynamic diameter (mm)	Polydispersity index (PDI)
1	RΔF	304 ± 20	0.07
2	MΔF	200 ± 15	0.12
3	VΔF	220 ± 25	0.22
4	FΔF	980 ± 45	0.36

**Fig. 1:**
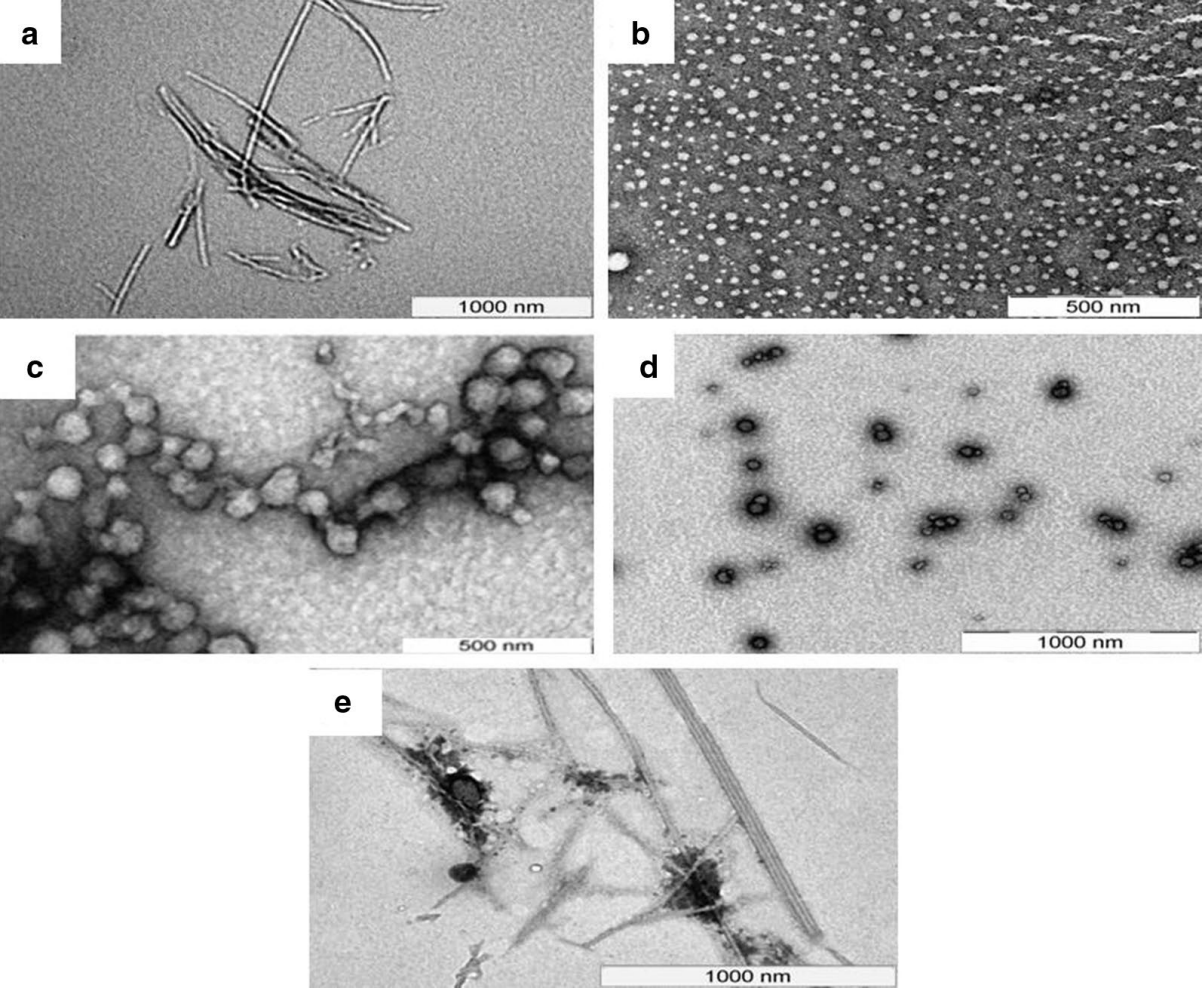
Transmission electron micrographs of DNPs: TEM image of **a** F∆F, showing the formation of tubular structure with mean diameter of 25 nm and length in microns, **b** M∆F, demonstrating the formation of vesicular structures with mean diameter of 40 nm **c** V∆F, showing the formation of vesicular structures with mean diameter of 55 nm, **d** RΔF demonstrating the formation of vesicular structures with mean diameter of 62 nm and **e** Ccm-F∆F showing dense tubular structures

Transmission electron microscop (TEM) was used to investigate the morphological details of the nanoparticles. It was observed that RΔF assembled into vesicular structures with mean diameter of 62 nm. M∆F and V∆F also formed vesicular structures with mean diameter of 40 and 55 nm respectively. However, similar to our earlier studies [41] the dipeptide F∆F under these conditions self-assembled into tubular structures with mean diameter of 25 nm and length in microns (Fig. 1a–d). The final properties of the peptide assemblies, including their size, shape are governed by a delicate balance of the intermolecular interactions mentioned above and hence different dipeptides form different types of nanostructures. Earlier studies have also demonstrated similar phenomenon where a slight change in peptide sequence resulted in the formation of nanostructures of varied morphologies and dimensions [42, 43].

### In vitro cytotoxicity and haemolytic assay

**Fig. 2:**
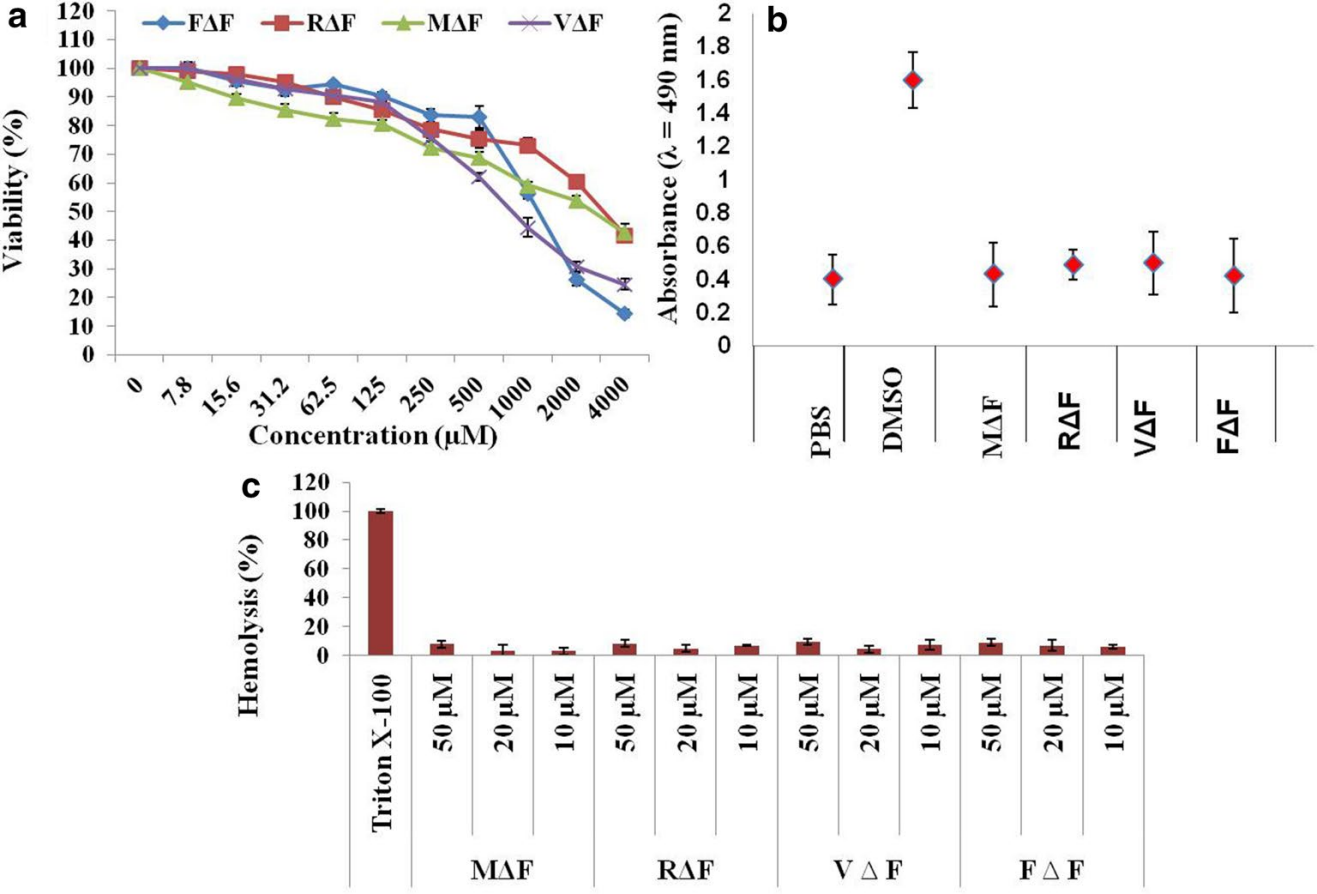
In vitro cytotoxicity and haemolytic assay: cell toxicity was assessed using **a** MTT assay. L929 cells were treated with different concentrations i.e., from 0 to 4000 µM of the DNPs for 24 h. Viability was expressed as the percentage of media control. **b** LDH release assay: cells treated with 50 µM of DNPs showed almost similar release of LDH like PBS treated cells. Cells treated with DMSO as positive control showed the maximum LDH release. **c** Percentage haemolysis at three different concentrations. None of the DNPs showed haemolytic activity. Triton X-100 taken as a positive control showed 100 % haemolysis

Nanoparticle based delivery systems offer several advantages like site-specific delivery of entrapped molecules, yet toxicity of nanomaterial towards healthy cells remains an important concern [19, 44]. In vitro cytotoxicity of void DNPs was assessed in mouse fibroblasts (L929) cultured in RPMI medium. Cells were seeded at a density of 1 × 10^4^ cells per well in 200 µl of cell growth medium and exposed to increasing concentrations of DNPs (0–4000 µM) for period of 24 h, followed by measurement of cell viability using MTT assay. The 50 % cytotoxicity concentration (CC_50_) of these DNPs were found to be 800, 1160, 2680 and 3100 μM for VΔF, FΔF, MΔF and RΔF respectively (Fig. 2a).

Cytotoxicity was also assessed with lactate dehydrogenase (LDH) release assay. LDH is a soluble cytosolic enzyme that is released into the culture medium following loss of membrane integrity resulting from either apoptosis or necrosis. LDH activity, therefore, can be used as an indicator of cell membrane integrity and serves as a general mean to assess cytotoxicity resulting from chemical compounds or environmental toxic factors. L929 cells treated with the DNPs at a concentration of 50 μM, exhibited similar release of LDH to the media as untreated cells, suggesting that these DNPs are safe for in vivo applications (Fig. 2b).

Nanoformulations delivered into the body, will finally enter the circulation and may adversely affect the red blood cells (RBCs). Hemolysis (destruction of red blood cells) can lead to anemia, jaundice and other pathological conditions; therefore the hemolytic potential of all intravenously administered pharmaceuticals must be evaluated. Measuring the percentage of hemolysis is an appropriate way to detect the toxicity of a test compound towards RBCs [45, 46]. We performed hemolytic assays for all four DNPs at three different concentrations (10, 20 and 50 µM) and found that none of the DNPs were hemolytic even at 50 µM concentration (Fig. 2c). Results of cytotoxicity and hemolysis assay suggested that these DNPs are highly biocompatible and therefore safe for biological applications, including intra venous drug delivery.

### Loading the DNPs with Ccm

Dissolution and entrapment of highly hydrophobic drugs like Ccm inside nano or micro-carriers requires strong nonaqueous solvents and it is generally difficult to remove the solvent from the final drug-nano formulation, which is a concern in their potential application in in vivo drug delivery [47, 48]. Dipeptide based nanoparticles reported here are prepared under largely aqueous environment endowing them the suitability for potential in vivo applications. After characterizing the DNPs, we next investigated their ability to load Ccm following the post loading method. Ccm was dissolved in methanol (10 mg/ml) and DNPs were incubated with three different concentrations of Ccm (1, 2 and 3 mg of Ccm/ml of DNPs) for 72 h. Unbound Ccm was removed by filtration (50 kDa Amicon^®^ Ultra-0.5) followed by lyophylisation of nanoforumlation. Optimum loading was observed at a Ccm concentration of 3 mg/ml. At this concentration, Ccm loading was found to be 68 ± 0.07 % (w/w) in F∆F nanotubes, ~8 % (w/w) for VΔF nanovesicles, ~12 % (w/w) for MΔF nanovesicles and ~14 % (w/w) for RΔF nanovesicles (Table 3). The relatively higher loading observed in case of FΔF could be attributed to optimum hydrophobic interactions between the drug molecules and the peptide nanotubes and also possibly to enhanced π–π interactions between planar aromatic structure of the drug and the two aromatic rings in FΔF. Since FΔF turned out to be most efficient in entrapping Ccm, this combination was taken up for further investigations. Ccm-FΔF analyzed using TEM demonstrated that Ccm loading did not disturb the overall morphology of the DNPs (Fig. 1e). lyophilised Ccm loaded FΔF nanotubes (Ccm-FΔF) were resupended in water and this suspension in colloidal form was used for drug release, stability as well as for in vitro and in vivo efficacy studies.

**Table 3:** Percentage loading of curcumin in DNPs at different concentrations: out of the four DNPs, FΔF showed highest loading at a curcumin concentration of 3 mg/ml

Curcumin (mg/ml)	% loading			
	FΔF	MΔF	VΔF	RΔF
1	42 ± 0.63	8 ± 0.33	6 ± 0.68	10 ± 0.72
2	55 ± 0.23	10 ± 0.13	7 ± 0.55	12 ± 0.12
3	68 ± 0.07	12 ± 0.66	8 ± 0.35	14 ± 0.76

### Release of Ccm from Ccm-FΔF

**Fig. 3:**
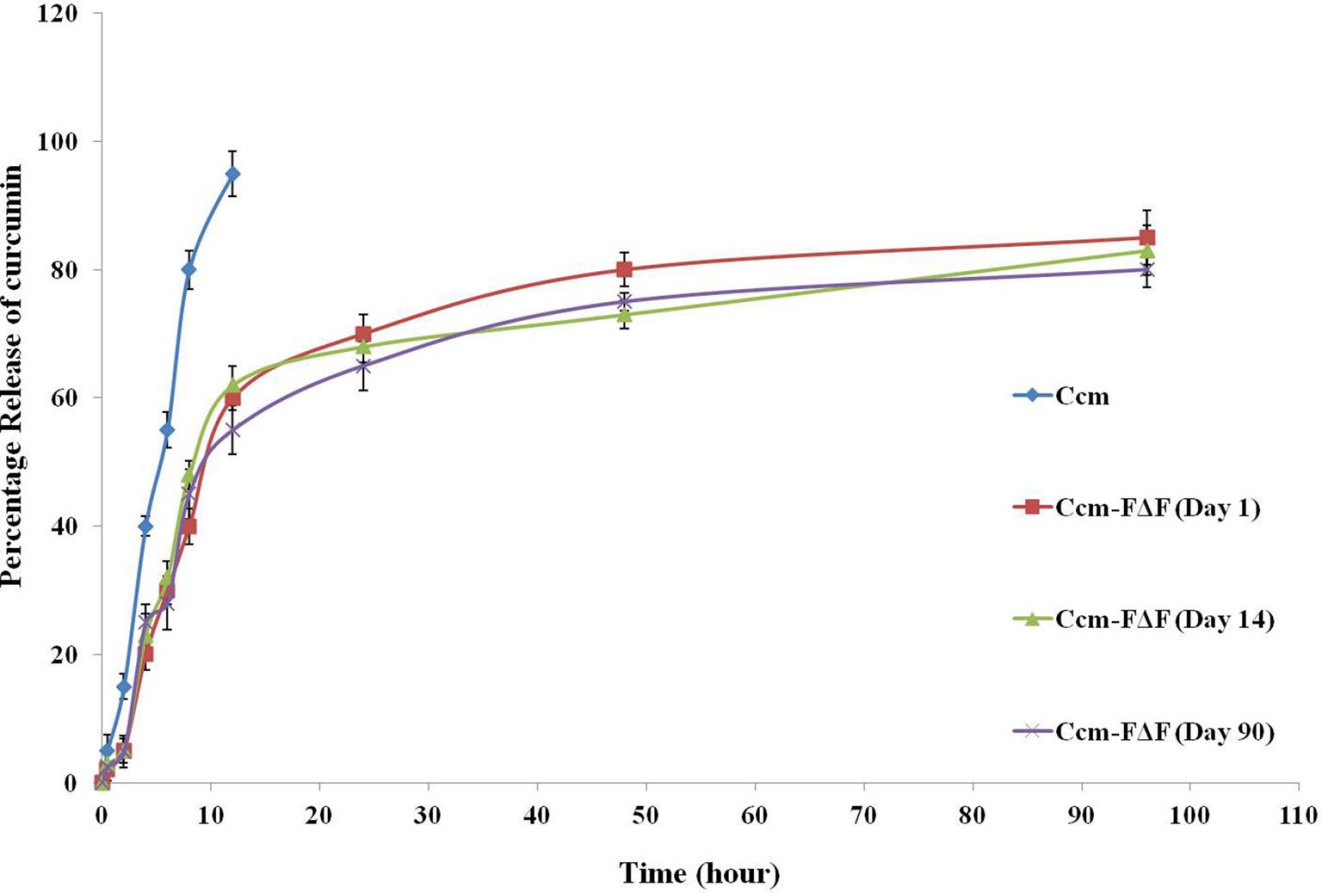
Release of curcumin from Ccm-FΔF: in vitro curcumin release from Ccm-FΔF nanoformulations, stored at room temperature for different time points (day 1, 14 and 90) in methanol: water (1:1 v/v). Curcumin content was estimated using (UV–Vis) spectrophotometer at a wavelength of 425 nm. (n = 3), *error bar* represent ± standard deviations

Ccm release from the Ccm-FΔF was followed by using dialysis membrane bag method [49, 50]. Release was monitored for a period of 96 h in a mixture of methanol–water (1:1 v/v) as dissolution media. Methanol–water (1:1 v/v) was used as a release media because of appropriate solubility of Ccm in this media which would maintain a sink like condition for the drug release [51]. Release patterns of both free and nanoparticle bound Ccm are shown in Fig. 3. Free Ccm showed a quicker release (55 ± 2.8 %) from the dialysis membrane as compared to Ccm-FΔF (30 ± 2.5 %) over a period of 6 h. Where almost 90 % of free Ccm was released from the dialysis membrane in just 9–10 h, the Ccm-FΔF nanoformulations took almost 90 h to release 80 % of the initial loaded Ccm concentration. Similar release pattern was also observed earlier in case of Ccm loaded in MAX8 peptide hydrogel [52].

### Stability of Ccm-FΔF nanoparticles

**Fig. 4:**
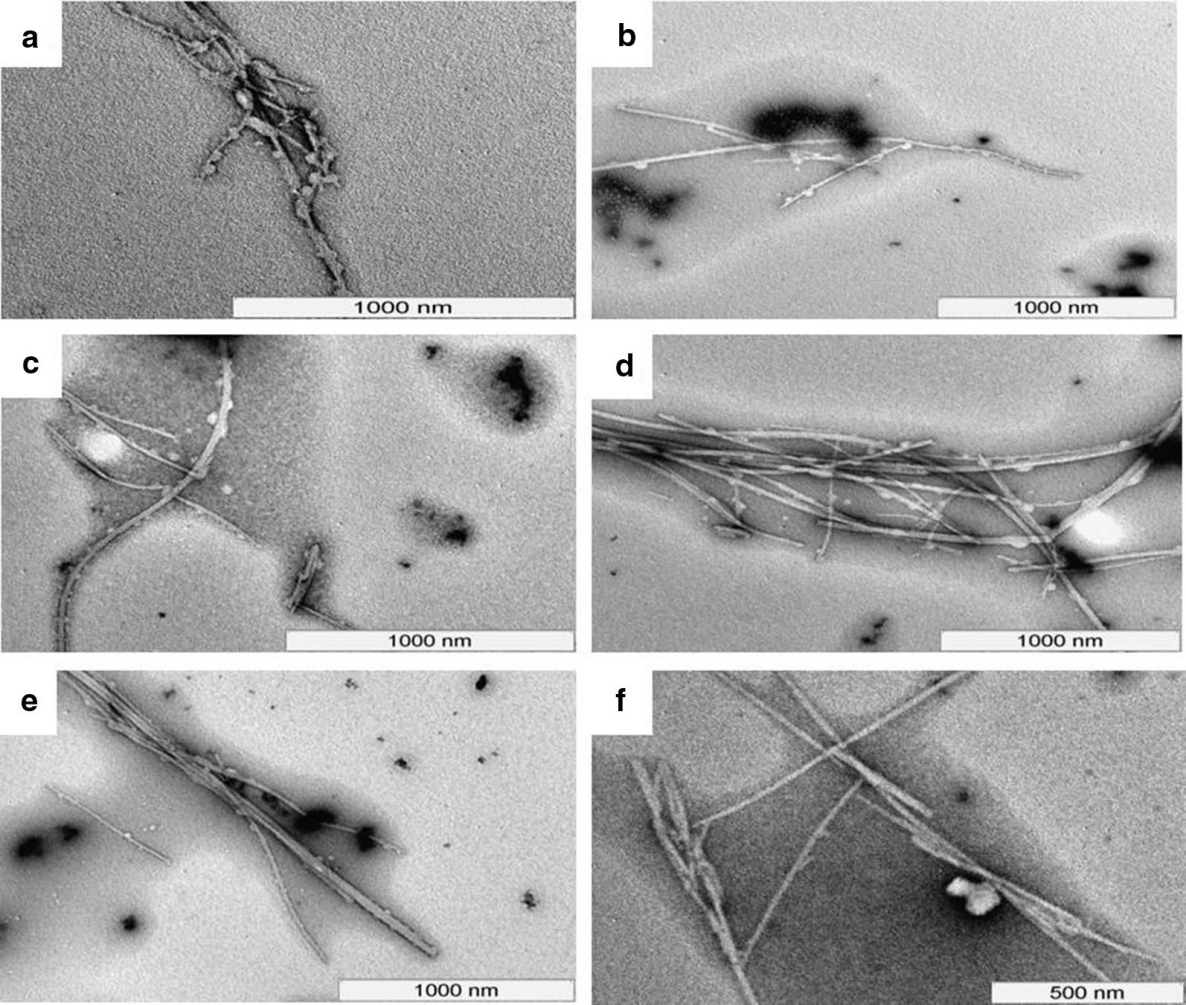
Stability of Ccm-FΔF nanotubes: TEM photographs of curcumin loaded nanotubes at different time points. **a**–**f** represent images taken after 1,7,14, 28, 56 and 90 days of incubation at room temperature (25 ± 2 °C). Results demonstrated the stability of curcumin loaded nanotubes over the entire incubation period of 90 days

Use of nanoparticle based systems as possible drug delivery agents necessitates them to be stable over a period of time [53]. We assessed the stability of Ccm-F∆F nanoparticles towards various parameters which include, morphological stability using TEM imaging, retention of drug content and drug release behavior. After being stored for different time periods (1, 7, 14, 28 and 56 and 90 days) at room temperature (25 ± 2 °C), Ccm-F∆F were resuspended in water (1 mg/ml) and observed under TEM. Results demonstrated that the morphology of Ccm-FΔF nanoparticles were intact even after 90 days of storage at room temperature ((25 ± 2 °C) Fig. 4).

**Fig. 5:**
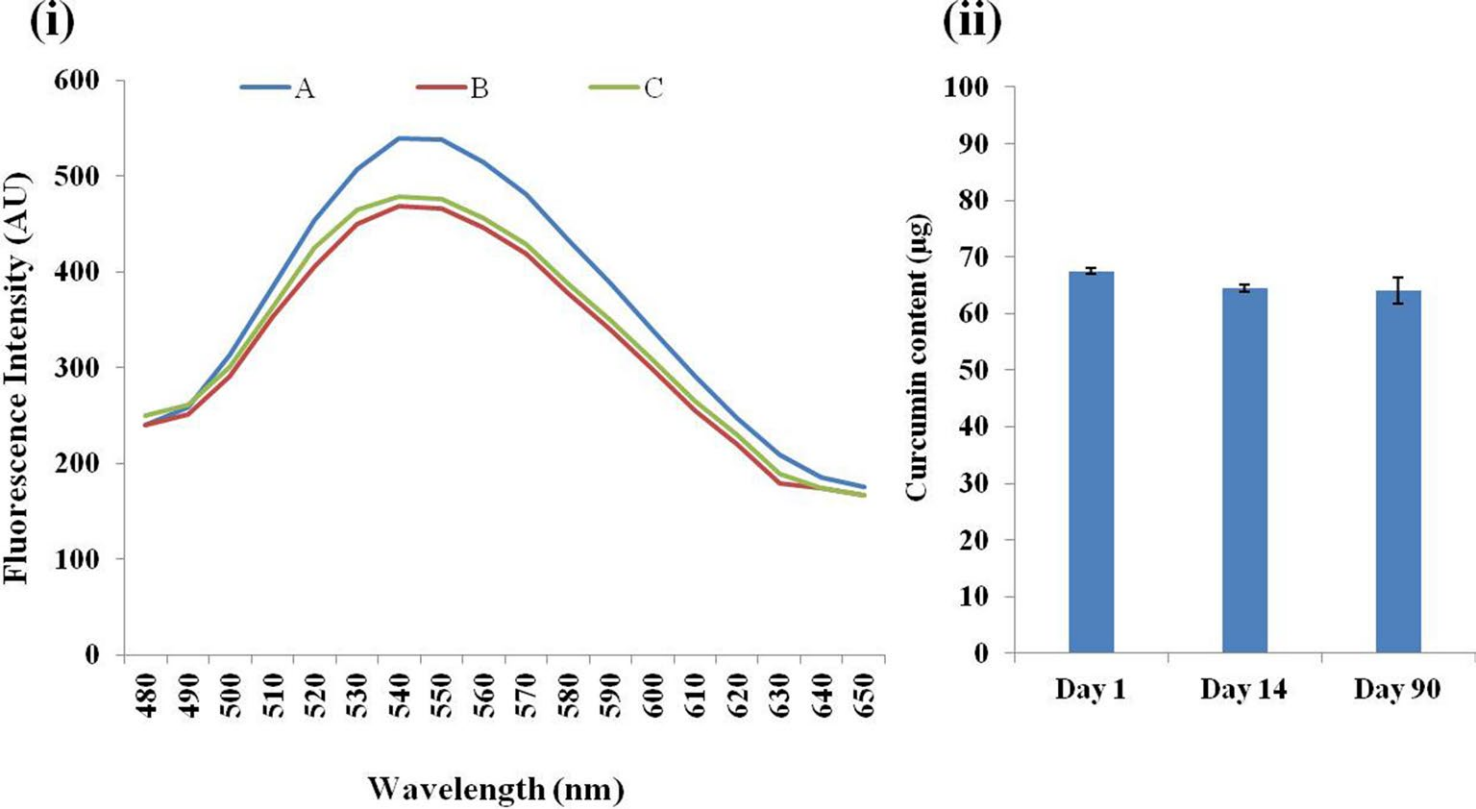
Fluorescence emission spectra of curcumin: **i** fluorescence spectra of both curcumin (*A*) and Ccm-FΔF at two different points, (*B*) at 1 day and (*C*) after 90 days, in aqueous solution of methanol (1:1, v/v) at an excitation wavelength of 425 nm. **ii** Amount of curumin present in Ccm-FΔF nano-formulations during the incubation period. Curcumin content was determined at three different time points (1, 14 and 90 days). It was observed that the curcumin concentration inside the nanotubes remained almost constant even after 90 days of storage depicting the stability of the drug inside the nanotubes

Stability of drug content in the nanoparicles was also proved by determining Ccm stability, content and release from Ccm-F∆F over the period of storage. Due to its polyphenolic structure, Ccm shows inherent fluorescence properties, which also depends on the molecules local environment [54]. Thus, the stability of Ccm inside the nanoparticles was determined by measuring its fluorescence properties. Fluorescence spectra of a methanol: water solution (1:1; v/v) of Ccm taken at an excitation wavelength of 425 nm showed an emission peak at 545 nm. An aqueous methanolic solution of Ccm-FΔF at two time points (day 1 and 90) showed a similar emission pattern, suggesting that entrapment in FΔF DNPs had no effect on the photophysical properties of Ccm and also the drug is stable inside the DNPs for a period of 90 days (Fig. 5i). We next estimated the Ccm content in the DNPs after being stored at the room temperature (25 ± 2 °C) for a period of 90 days and found that there was no significant change in the Ccm content of the nanoformulations during these time period (Fig. 5ii). Further, Ccm-FΔF nanoformulations stored at room temperature (25 ± 2 °C) for 90 days showed release patterns similar to that found on day 1 (Fig. 3). These results taken together demonstrate long term stability of the Ccm-FΔF nanoparticles as well as the drug, Ccm, in the DNPs.

### In vitro antimalarial activity

**Fig. 6:**
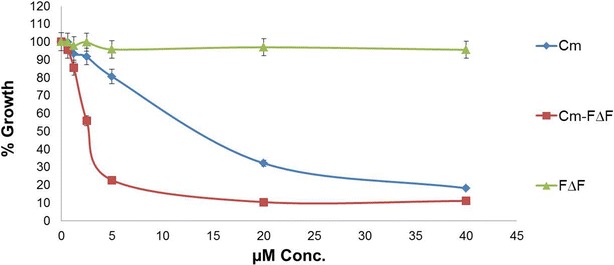
Malaria parasite (*Pf indo*) inhibition assays under in vitro *conditions*: Curcumin entrapped in nanotubes inhibited the growth of chloroquine-resistant *P.falciparum* (*Pf indo*) in culture, more efficiently (IC_50_, 3 µM) than free curcumin (IC_50_, 13 µM). Void nanotubes (F∆F) did not show any inhibitory effect

Antimalarial activity of Ccm has been reported previously [16, 17]. However, its poor aqueous solubility and hence low bioavailability has limited its use as an anti-malarial agent. We have addressed this issue by loading Ccm in the DNPs and evaluated their activity in parasite culture. Ccm-F∆F showed significant growth inhibition against chloroquine resistant *P.falciparum* (indo) in comparison with the free drug and nanoparticles alone (Fig. 6). The IC_50_ value of Ccm-F∆F nanoparticles and free Ccm against *P. falciparum* was found to be 3.0 and 13 µM respectively. Thus, the drug loaded nanoparticles demonstrated almost fourfold reduction in the IC_50_ concentration as compared to native Ccm. Enhancement in anti-malarial activity was also found in case of Ccm loaded in chitosan nanoparticles [55].

### In vivo antimalarial activity of Ccm-FΔF

**Fig. 7:**
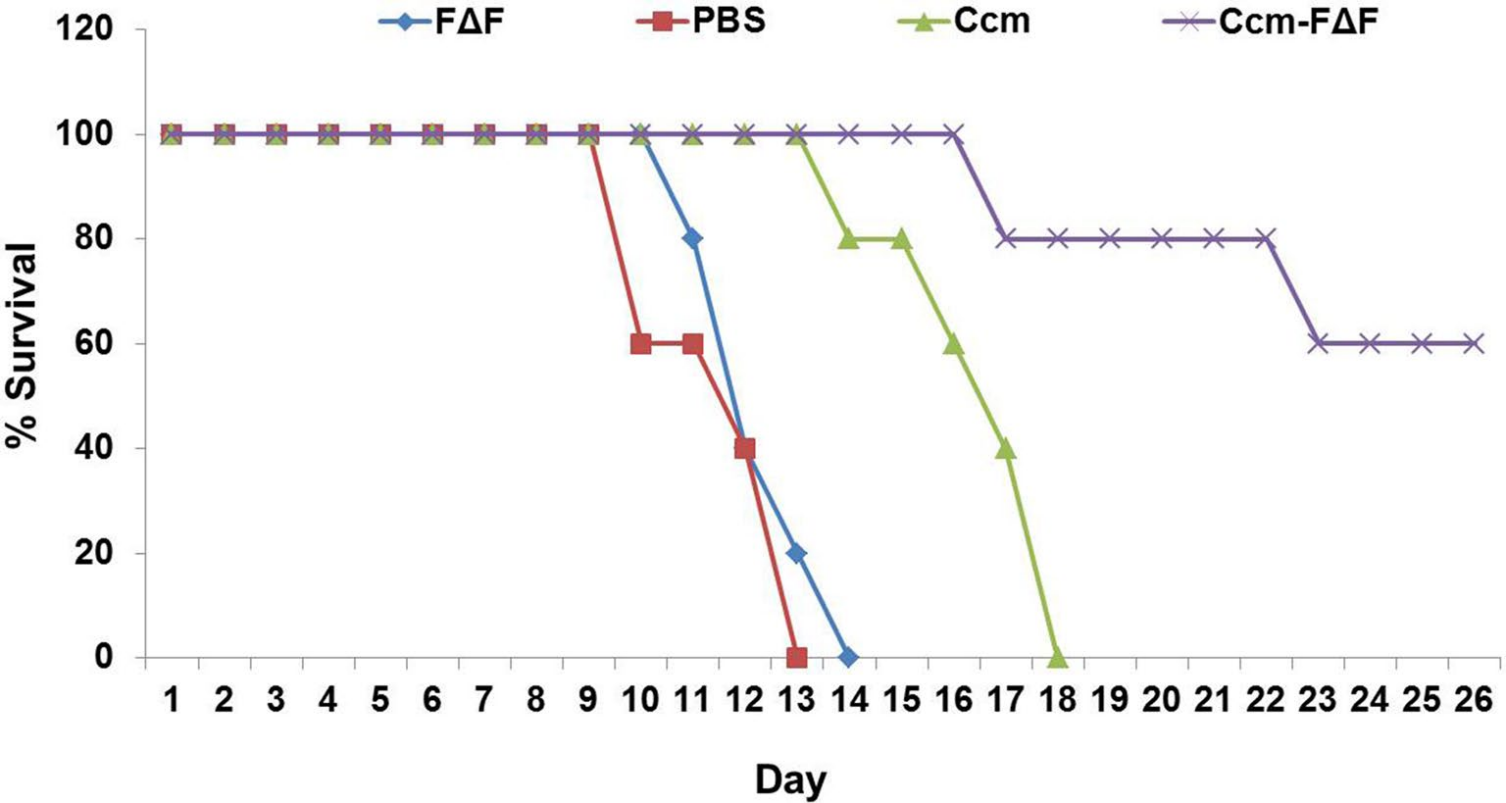
Survival graph of *P. bergi*-infected mice treated with different groups. Most of the mice in the group treated with PBS and FΔF died with high parasitemia between 10 and 14 days of infection. Mice treated with free Ccm showed increased life span but died earlier than those treated with Ccm-FΔF

**Fig. 8:**
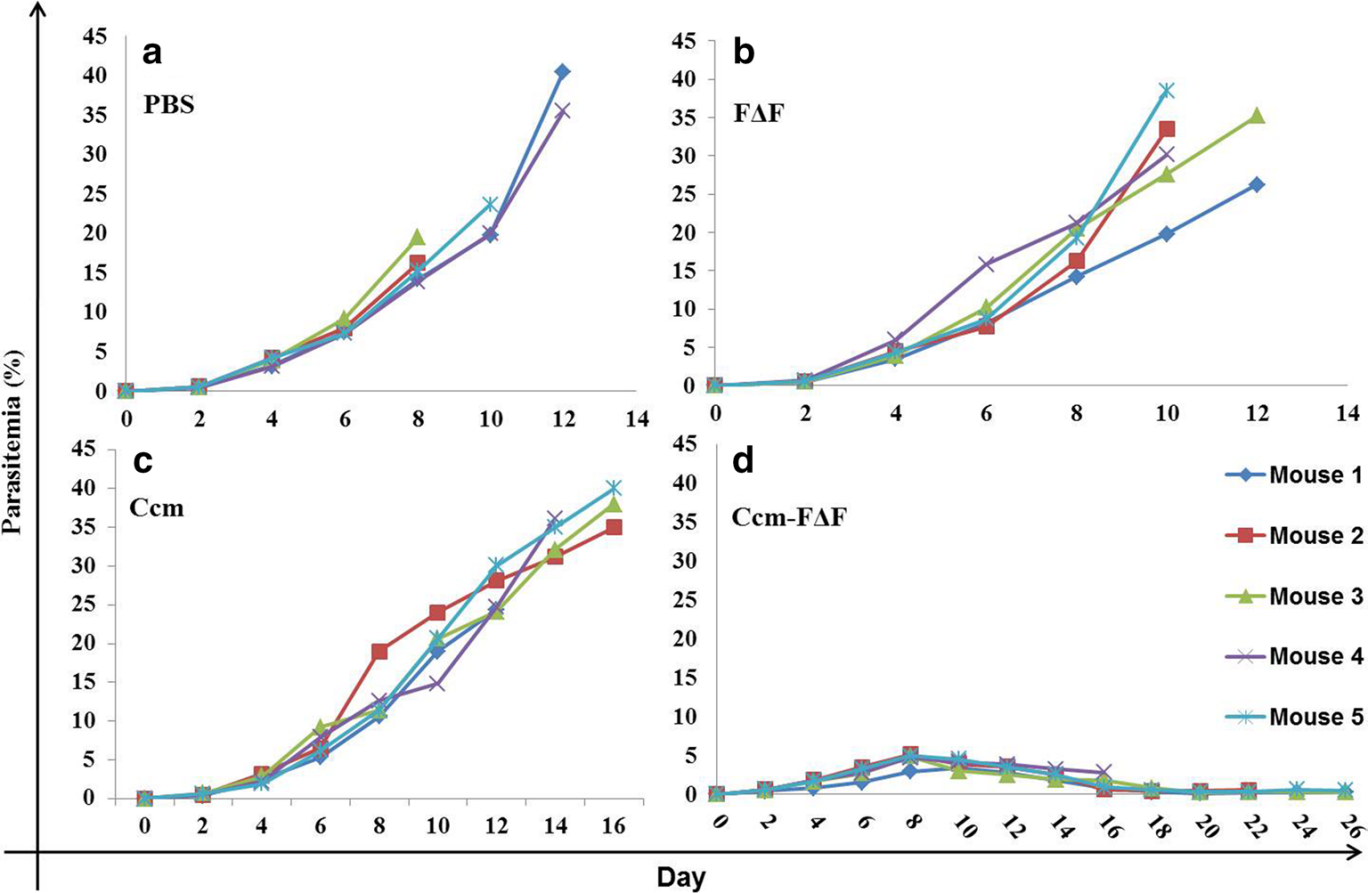
Percentage parasitemia of different groups of mice: Mice treated with intra-peritoneal injection of the nanoformulations. **a** PBS and **b** FΔF treated group. These groups showed increase in parasitemia with time killing all the animals. **c** Ccm (50 mg/kg BW of curcumin) treated group, where parasitemia rose slowly and mice survived for the longer time as compared to the PBS control group. **d** Mice treated with Ccm-FΔF (equivalent to 50 mg/kg BW of curcumin) showed significant decrease in parasitemia and increase in life span

In vivo antimalarial efficacy of Ccm-FΔF nanoparticles and free Ccm in *P. berghei (ANKA)* infected BALB/c mice is presented in Figs. 7 and 8. An analysis of the survival graph of mice and parasite growth count analysis revealed differences between the control groups versus the groups treated with free and entrapped Ccm. In this assay, all mice in the control group treated with PBS, and FΔF, died with high parasitemia between day 10 and 14. In the group of mice treated with Ccm alone also parasitemia rose at the same rate as the control group, although mice in the group survived somewhat longer than the PBS control group, all mice in this group died by day 18. In mice treated with Ccm-FΔF, there was a significant reduction in the growth of parasitemia. Animals administered with Ccm-FΔF showed an increased life span and enhanced survival rate as compared to those treated with Ccm alone. Earlier studies have shown that curcuminoids have beneficial therapeutic effects only in their active form [56, 57]. The attenuated effect of free Ccm might be due to immediate degradation of curcuminoids to inactive metabolic products (*trans*-6-(4-hydroxy-3-methoxyphenyl)-2,4-dioxo-5-hexenal, vanillin, ferulic acid and feruloyl methane) in the blood. The slow release mechanism of Ccm from the nanoparticles as evident from the in vitro release studies was expected to maintain an effective concentration of Ccm in the blood as compared to free Ccm leading to an enhancement in parasite killing efficacy.

## Conclusions

Here, we have described the synthesis and characterisation of Ccm loaded self-assembled DNPs, which can be easily prepared under relatively mild aqueous conditions. These DNPs are non-cytotoxic and non-haemolytic. Ccm loaded DNPs (Ccm-FΔF) showed much higher activity of Ccm in comparison to free Ccm under both in vitro and in vivo conditions. Such short peptide based delivery systems may have potential for further development for applications in the field of malarial drug delivery.

## Methods

*N*-methyl morpholine (NMM), 1,1,1,3,3,3-hexafluoro-2-propanol (HFIP), trifluoroacetic acid (TFA), Ccm (diferuloyl methane), isobutyl chloroformate (IBCF), methanol and DL-threo-β-Phenylserine was purchased from Sigma–Aldrich (St. Louis, MO, USA). N-[(*tert*-butoxy)carbonyl]-l-methionine, l-phenylalanine, Anhydrous sodium sulfate and citric acid were obtained from Novabiochem (Merck, Darmstadt, Germany). Diethyl ether, Sodium acetate, tetrahydrofuran (THF), ethyl acetate, and acetonitrile were purchased from Spectrochem Pvt Ltd (Mumbai, India). Cell lines L-929 (Mouse fibroblast) from ATCC (Manassas, VA). Chloroquine resistant strains of *P*. *falciparum* (Indo) were obtained and grown in human O^+^ erythrocytes at 3 % hematocrit in a complete medium (RPMI 1640 medium supplemented with 25 mM HEPES, pH 7.5, 25 mM sodium bicarbonate, 50 mg/liter hypoxanthine,0.5 % Albumax II, and 40 µg/ml gentamicin sulfate). Cultures were maintained at 37 °C in a gas mixture of 5 % CO_2_ and 3 % O_2_. All other chemicals and buffers were of the highest grade available.

### Synthesis of nanostructure forming dipeptides

Synthesis of phenylalanine-dehydrophenylalanine (FΔF), was carried out using solution phase peptide synthesis. In brief, Boc-Phe-OH (10 mM; 2.48 g) was dissolved in anhydrous THF, the solution was chilled to −20 °C in an ice-salt mixture and kept for stirring for 10 min. IBCF (10 mM; 1.39 ml) was then added to the solution followed by NMM (10 mM; 1.31 ml). After stirring for 20 min, a pre chilled solution of DL-threo-β-phenylserine (11 mM; 1.98 g) and sodium hydroxide (11 mM; 0.44 g) in MQ-water were added. Reaction mixture was stirred over night at room temperature and concentrated in rota evaporator. The residual solution was acidified with chilled concentrated solution of citric acid. Extraction of intermediate product (Boc-Phe-DL-threo-β-phenylserine) was done by using ethyl acetate. Ethyl acetate solution was then dried by passing through anhydrous sodium sulphate followed by drying in rota vapour. Dried Boc-Phe-DL-threo-β-phenyl serine were solubilised in acetic anhydride (100 ml) and mixed with sodium acetate (6.5 mM; 1.16 g) and stirred for 36 h. Reaction was stopped by adding crushed ice and filtered the precipitate using grade four filtered funnel. Filtrate was washed thrice with cold water and dried in desiccators. Obtained powder (Boc-Phe-ΔPhe-azalactone) was dissolved in methanol and stirred with 1.5 equivalent of 1 N NaOH for 4 h. Resulting solution was concentrated on rota vapour and extracted with ethyl acetate. Peptide was de protected by treating the compound with anhydrous tetrahydrofuran (THF) and purified on reverse phase HPLC(LC-6 AD, Shimadzu, Kyoto, Japan) using C18 column (Phenomenex, Hyderabad, India, C18, 5 μm, id 250 × 4.6 mm) in acetonenitrile (0.1 % TFA)-water (0.1 % TFA) with 2 % linear gradient. Other di-peptides used for this study were synthesised using similar method as described above. The mass of peptide were obtained by using mass spectrometer (AppliedBiosystemsQStar (Q-TOF), Ontario, Canada).

### Preparation and characterization of DNPs

Nanostructure of different dipeptides was prepared by dissolving the dipeptides (2 mg each in case of V∆F, M∆F, R∆F and 0.5 mg in case of F∆F) in 100 μl of isopropanol. While MΔF and RΔF were found to be soluble in isopropanol, VΔF and FΔF were only soluble when heated. Self-assembly of these dipeptides was initiated by addition of 1 ml of water to the isopropanol solution of the dipeptides followed by incubation for 4–6 h at room temperature prior to use.

### Dynamic light scattering studies

DLS was used to determine the particle size and size distribution of these self-assembled nanoparticles. Light scattering studies were performed on Zetasizer NanoZS90 (Malvern Ltd, Malvern, UK) at an angle of 90° using a 633 nm laser. All these experiments were performed at room temperature and under dust free environment.

### Structural characterization of DNPs using transmission electron microscopy

Transmission electron microscopy of DNPs was carried out using uranyl acetate negative staining method. In brief, DNPs were loaded by adsorbing a drop of DNPs on a 200 mesh 3 mm carbon supported nickel grid. Staining of loaded DNPs was done by incubating the loaded grid with 1 % uranyl acetate for 30 s at room temperature. The loaded grid was air dried at room temperature prior to be observed under the microscope. Samples were observed under a transmission electron microscope (TEM) (Tecnai 120 BioTWIN, FEI Netherlands) operated at 120 kV. The image was captured using a Megaview II digital camera and analysis was carried out using Analysis II (Megaview, SIS, Germany) the iTem software package.

### In vitro cytotoxicity assays

#### Cell viability assay (MTT assay)

Cytotoxic effect of void DNPs was assessed by the 3-(4,5-dimethylthiazole-2-yl)-2,5-diphenyltetrazolium bromide (MTT) dye conversion assay. MTT is a yellow tetrazole that gets converted into a purple insoluble formazan because of the mitochondrial reductase enzyme present in live cells. The Mouse fibroblast cell line (L929) was purchased from ATCC and maintained in Roswell Park Memorial Institute (RPMI) cell growth medium supplemented with 10 % heat inactivated fetal bovine serum (HI-FBS) at 37 °C in a 5 % CO_2_ incubator. Following two passages, the cells were harvested and seeded at a density of 1 × 10^4^ cells/well in 200-μl of complete cell culture growth medium, in a 96-well cell culture plate. After 12 h of incubation, the cultured cells were treated with different concentrations (0–4000 µM) of DNPs and incubated in the same incubator for another 24 h. Media was then replaced with fresh media after 24 h and cells were treated with 20 µl (5.0 mg/ml in PBS) of MTT (filter sterilised using 0.2 µ filter) for 4 h. Once the incubation period was over, media from each well was removed and 100 μl of DMSO was added into each well to dissolve purple formazan which is formed in live cells. The absorbance of formazan was measured at 570 nm using a microplate reader (VERSAmax Tunable Microplate Reader; Molecular Devices, CA, USA). The cell viability was expressed as the percentage of control using the following equation:

$${\mathbf{Percentage}}\, \left( \% \right) \,{\mathbf{viability}} \,{\mathbf{of}}\, {\mathbf{cells}} = \frac{{{\mathbf{Abs}}\, ({\mathbf{T}})}}{{{\mathbf{Abs}}\, ({\mathbf{C}})}} \times {\mathbf{100}}$$

where “Abs (T)” is the absorbance of cells treated with DNPs and “Abs (C)” is the absorbance of the untreated cells.

#### Lactate dehydrogenase (LDH) leakage assay

Lactate dehydrogenase is a cytosolic enzyme present in live cells. In case of any damage to plasma membrane LDH is extruded into the media. Quantitative measurement of LDH leakage provides an estimate of the cellular cytotoxicity caused due to loss of membrane integrity. LDH activity in the supernatant of the culture medium was determined using a commercial LDH based in vitro toxicology assay test (TOX-7, sigma). In short, 1 × 10^4^ cells were seeded into each well of a 96-well cell culture plate containing 200 µl of complete growth medium. After 12 h of incubation at 37 °C temperature in 5 % CO_2_, cells were treated with threefold higher concentration i.e. 50 µM of different DNPs and with DMSO as positive control. After 24 h treatment period, the culture plate was centrifuged in a swing bucket rotor at 250*g* for 5 min at 37 °C. Fifty micro-liters of supernatant was taken in a 96 well plate and mixed with equal volume of LDH mixture and incubated at room temperature for 30 min. Quantification of the LDH release into the media was performed by measuring the absorbance at 490 nm.

#### Haemolysis assay

Haemolysis assays were performed in heparinised whole blood (2.5 ml) obtained from a healthy male human volunteer. The blood sample was centrifuged at 1000×*g* for 20 min at 37 °C, buffy coat (white blood cells) was removed and the packed cells were washed twice with sterile PBS. PBS was added to the RBCs to obtain 2 % haematocrit. One-hundred micro-liters of cell suspension was added to each well of a 96 well plate containing different concentrations of the DNPs. Negative control included 50 µl of PBS solutions added to 100 µl of cell suspension (as red blood cell do not lyse in isotonic condition) and 50 µl of 1 % Triton X-100 was added in another well as the positive control (as RBCs lyse in a hypotonic medium). Void DNPs of three different concentrations (10, 20 and 50 µM) were added to 100 µl of cell suspension. Samples were incubated at 37 °C for 60 min. The reaction was stopped by addition of 50 µl of 2.5 % glutaraldehyde. Blood samples were then centrifuged at 1000×*g* for 15 min at 37 °C and the absorbance of the supernatant was measured at 540 nm using UV–Vis spectrometer. The percentage haemolysis was calculated using the following equation:

$${\mathbf{Hemolysis}}\, (\% ) = \frac{{{\mathbf{Absorbance}}\, {\mathbf{of}} \,{\mathbf{Ts}} }}{{{\mathbf{Absorbance}}\, {\mathbf{of}}\, {\mathbf{Pc}} }} \times {\mathbf{100}}$$

where “Ts” is the absorbance of RBC treated with different concentrations of DNPs and PBS. Whereas “Pc” is the absorbance of RBC treated with 1 % Triton X-100.

### Loading of Ccm in DNPs

Loading is the most important benchmark for measuring the nanoparticle efficacy as a drug delivery vehicle. Following the formation of nanoparticles (as discussed above), Ccm was loaded on to the DNPs by following post loading method. Briefly, Ccm was added to the DNPs from a stock solution of the drug (10 mg/ml in methanol), at three different concentrations i.e. 1, 2 and 3 mg/ml of the DNPs and incubated at room temperature for 72 h with gentle shaking. These nanoparticle drug formulations were further processed by ultrasonication for three minutes using sonication probe to remove any large aggregates formed. The nanoparticle dispersions were washed twice with filtered deionized water by centrifuging at 600×*g* for 30 min at room temperature, shock-frozen in liquid nitrogen and lyophilised at 0.40 m bar and −80 °C for 24 h using freeze dryer. The lyophilised powder was then resuspended in 1 mg/ml filtered deionized water and characterised using DLS and TEM. To determine the percentage loading capacity (LC) of Ccm in the DNPs, we employed methods described in previous studies [36]. In brief, lyophilized Ccm-loaded DNPs were dissolved in 1 ml of methanol. The samples were then centrifuged at 3300*g* for 30 min at room temperature. The amount of Ccm in the supernatant was determined at 425 nm using V–Vis spectrophotometer. Calibration curve was generated using the reference standard and the loading capacity was determined as follows.

$$\% \, {\mathbf{Loading}} \, {\mathbf{of}} \, {\mathbf{curcumin}} = \, \left( {{\mathbf{W}}_{{{\mathbf{np}}}} / \, {\mathbf{W}}_{{{\mathbf{ad}}}} + {\mathbf{W}}_{{{\mathbf{dp}}}} } \right) \, \times \, {\mathbf{100}}$$

where W_np_ refers to the total weight of Ccm in the nanoparticles; W_ad_ the weight of Ccm added to the nanoparticles and W_dp_ the total dipeptide weight in the formulation.

### In vitro Ccm release

An In vitro release study of Ccm was performed to monitor the Ccm release profile at different time points using dialysis bags (MWCO: 3000) with floater (Spectrum Laboratories, CA, USA). Briefly, lyophilised Ccm-FΔF nanoparticles (Stored for different time points day1, 14 and 90) equivalent to 1 mg of Ccm, was dispersed in 1X PBS and filled in a dialysis bag, stirred at 100 rpm at 37 °C, under sink condition in 250 ml of 1:1 methanol: water, due to higher solubility of Ccm in this solvent mixture. At different time points (0.5, 1, 2, 4, 6, 12, 24, 48, and 96 h) 1 ml of receptor medium was removed and replaced with same volume of fresh medium to maintain the total volume of release medium. To estimate Ccm release, 1 ml of receptor medium was lyophilised, resuspended in methanol and quantified using (UV–Vis) spectrophotometer at a wavelength of 425 nm.

### Long term stability of Ccm loaded FΔF (Ccm-FΔF)

Long term stability of Ccm-FΔF was analyzed by a FEI Tecnai TEM at 120 kV (FEI Europe, The Netherlands) at different (1, 7th, 14th, 28th and 56th and 90th day) time points after negative staining with uranyl acetate (1 % in MQ water). Nanoformulations were prepared and lyophilised as described above, resuspended in PBS and stored at room temperature, to evaluate the long term stability. On completion of different time points (1, 7th, 14th, 28th and 56th and 90th day) Ccm-FΔF were adsorbed on 200 mesh 3 mm carbon supported nickel grids and stained for 30 s with 1 % uranyl acetate and viewed under the electron microscope. Photomicrographs were digitally recorded using a Megaview II (SIS, Germany) digital camera. Image analysis to measure tube dimensions was carried out using Analysis II (Megaview, SIS, Germany) software package.

### Photophysical properties of Ccm and Ccm content in Ccm-FΔF

To determine whether entrapment in DNPs had any effect on Ccm’s photophysical properties after a long term storage of 90 days at room temperature (25 ± 2 °C), fluorescence spectra of Ccm-FΔF were taken at different time points (day 1 and 90) and compared with that of free Ccm. Fluorescence spectra of both native Ccm and Ccm-FΔF at a concentration of 1 mg/ml were measured in an aqueous solution of methanol (1:1 v/v, methanol: water). Fluorescence emission spectra were recorded from 480 to 650 nm with an excitation wavelength of 425 nm (LS 55; Perkin Elmer). To determine the stability of Ccm in the nanoparticles without any leakage, total Ccm content in the DNPs was checked at different time points (day 1, 14 and 90). This was carried out by dissolving 100 µg of Ccm-FΔF in 500 µl of methanol and then recording the absorption spectra at 425 nm using a microplate reader (VERSA max Tunable Microplate Reader; Molecular Devices, CA, USA). Ccm content was determined by comparing the sample with Ccm standard curve in methanol.

### In vitro anti-malarial activity of Ccm-FΔF

Chloroquine resistant *P. falciparum* INDO strain was used for growth inhibition assays. Parasites were grown under in vitro conditions by the method of Trager and Jensen with minor modifications [58]. In short, parasite cultures were maintained in fresh O^+^ human erythrocytes with 4 % hematocrit in complete RPMI1640 medium (RPMI 1640 with 0.2 % sodium bicarbonate, 0.5 % Albumax, 45 mg/l, hypoxanthine and 50 mg/l gentamicin) at 37 °C under reduced O_2_ (gas mixture of 5 % O_2_, 5 % CO_2_, and 90 % N_2_). Ccm, curcumin loaded FΔF (Ccm-FΔF) and FΔF stocks were prepared in filtered sterile water. The stocks were diluted to get final assay concentrations (0–40 µM/100 µl of complete media) and transferred to sterile 96-well flat-bottom tissue culture grade plates. Plasmodium cultures were synchronized at ring stage by 5 % sorbitol solution. Synchronized culture was transferred to drug containing 96-well plates at 2 % hematocrit and 1 % parasitemia. Plasmodium growth inhibition was measured by carrying out high throughput fluorescence based SYBR Green I assay. After 48 h of incubation the fluorescence of the samples were determined using a 96-well fluorescence plate reader (Victor, Perkin Elmer), with excitation and emission wavelengths at 485 and 530 nm, respectively. The fluorescence readings were plotted against drug concentration and IC_50_ values were calculated.

### Determination of in vivo efficacy

#### Malarial parasites

The rodent malarial parasite, *P. berghei* ANKA strain was maintained in BALB/c mice by weekly passage of infected blood containing 1 × 10^5^ parasites intraperitonially (i.p.).

#### Animal model

Male mice (BALB/c), 4–6 weeks old, weighing 18–20 g were housed in the animal maintenance facility of International Centre of Genetic Engineering and Biotechnology, New Delhi, India. Animal experiments were approved by the Institutional Animal Care and Use Committee and animals were housed for a week for acclimatization in groups of five in the animal house. The animals were fed on commercial pellet diet and water ad libitum in glass bottles. They were maintained under standard conditions of humidity (55–60 %), temperature (22 ± 3 °C) and light (12:12 h light/dark cycles). Animals used in this study were healthy and did not show any pathological symptoms.

#### In vivo anti-malarial activity

**Fig. 9:**
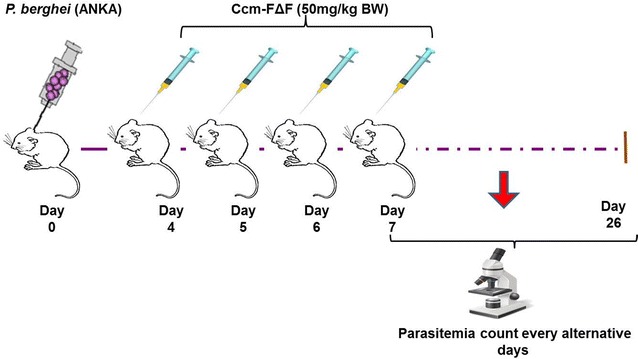
In vivo antimalarial assay design. After infection with *P. berghei* (ANKA) mice were treated with different formulations in the corresponding group and parasitemia count was determined every alternative day

Malaria was induced in BALB/c mice (with 18–20 g body weight) through blood transfusion. ANKA strain of *P.bergi* red blood cells (pRBC) were taken from an infected donor BALB/c mouse (10 % parasitimia) and diluted in PBS to 5 × 10^7^pRBC/ml. Mice were infected intraperitoneal with an aliquot of 0.2 ml of this suspension. Mice were then randomly divided into four groups with five mice in each group. Group I: *P. berghei* infection with PBS treatment; Group II: *P. berghei* infection and FΔF treatment; Group III: *P. berghei* infection and treatment with Ccm-FΔF (actual Ccm content was 50 mg/kg body weight); Group IV: *P. berghei* infection and Ccm treatment (50 mg/kg body weight). After reaching paracitemia 1–2 %, mice were treated for four successive days by intraperitoneal injection. Blood smears were prepared every alternate day from the tail vein for the period of 26 days (Fig. 9). Animals were kept under daily supervision for clinical signs and weight loss.

#### Measurement of parasitemia

Measurement of parasitemia was carried out after giemsa staining of parasites followed by microscopic imaging under oil immersion objective at 100× magnification. The percentage of infected erythrocytes was calculated in the fields of 1000 erythrocytes.

## Abbreviations

FΔF: phenylalanine–α,β-dehydrophenylalanine
RΔF: arginine-α,β-dehydrophenylalanine
VΔF: valine-α,β-dehydrophenylalanine
MΔF: methonine-α,β-dehydrophenylalanine
Ccm-F∆F: curcumin loaded F∆F nanotubes
DLS: dynamic light scattering
TEM: transmission electron microscope
PLGA: poly (lactic-co-glycolic acid)
DNPs: dipeptide nanoparticles
MWCO: molecular weight cut-off
IC_50_: inhibitory concentration
IP: intra peritoneal
Ccm: curcumin
kV: kilo volt
